# NLRP3-Dependent Pyroptosis: A Candidate Therapeutic Target for Depression

**DOI:** 10.3389/fncel.2022.863426

**Published:** 2022-05-26

**Authors:** Teng Wan, Xiaoyu Li, Mingyuan Fu, Xiaoyu Gao, Peiling Li, Weiming Guo

**Affiliations:** ^1^Sports Medicine Department, Huazhong University of Science and Technology Union Shenzhen Hospital, Shenzhen, China; ^2^The 6th Affiliated Hospital of Shenzhen University Health Science Center, Shenzhen, China; ^3^Hengyang Medical College, University of South China, Hengyang, China

**Keywords:** depression, pyroptosis, NLRP3, GSDMD, neuroinflammation

## Abstract

Depression, a major public health problem, imposes a significant economic burden on society. Recent studies have gradually unveiled the important role of neuroinflammation in the pathogenesis of depression. Pyroptosis, a programmed cell death mediated by Gasdermins (GSDMs), is also considered to be an inflammatory cell death with links to inflammation. Pyroptosis has emerged as an important pathological mechanism in several neurological diseases and has been found to be involved in several neuroinflammatory-related diseases. A variety of chemical agents and natural products have been found to be capable of exerting therapeutic effects by modulating pyroptosis. Studies have shown that depression is closely associated with pyroptosis and the induced neuroinflammation of relevant brain regions, such as the hippocampus, amygdala, prefrontal cortex neurons, etc., in which the nucleotide-binding oligomerization domain-like receptor protein 3 inflammasome plays a crucial role. This article provides a timely review of recent findings on the activation and regulation of pyroptosis in relation to depression.

## Introduction

Depression is a common disabling mental illness. Human susceptibility to depression has changed with variations in lifestyles, such as diet and sleep, and increased social stress (Bassi et al., 2020; Marx et al., 2021; Sweetman et al., 2021). Affluent environments will reduce depressive manifestations, while the majority of depressed patients in low- and middle-income countries do not receive effective treatment (Evans-Lacko et al., 2018; Gu et al., 2021). This imposes a heavy burden on individuals, families, and society. Until now, studies have shown that the onset and progression of depression are mainly related to neurotrophic support, disorders of monoamine neurotransmitters, dysregulation of excitatory and inhibitory neurotransmitters, and peripheral or central neuroinflammation (Prévot and Sibille, 2021; Tian H. et al., 2021). Recent studies have gradually unveiled the important role played by neuroinflammation in the pathogenesis of depression (Ali et al., 2020). Therapeutic strategies for neuroinflammation-related depression are developing continuously and providing promising results.

Pyroptosis is a programmed cell death mediated by Gasdermins (GSDMs), which has been discovered in recent years (Shi et al., 2017). In response to stimulation by viral and bacterial antigens and cytotoxins, inflammasomes are activated, leading to activation of downstream caspase-1. The downstream GSDM-N terminus, which is activated by caspase-1 shearing, eventually forms small pores in the cell membrane and leads to pyroptosis (Shi et al., 2017). Pyroptosis is closely related to tumor growth. It has been shown that the programmed death-ligand 1 has a non-immune checkpoint function and induces pyroptosis, thereby promoting tumor cell death (Wang et al., 2017; Hou et al., 2020). In addition, pyroptosis is also considered as a form of inflammatory cell death (Broz et al., 2020). Activation of caspase-1 cleaves both pro-interleukin (IL)-1β and pro-IL-18 and promotes their maturation in addition to the formation of GSDMD-induced pore channels. Eventually, these inflammatory factors are released extracellularly and induce inflammation (Broz et al., 2020). This property has contributed to the association of pyroptosis with a variety of inflammatory-related diseases, including infectious diseases and inflammatory diseases of the digestive and urinary systems (Man et al., 2017; Xu et al., 2018; Lin et al., 2020). Pyroptosis has also been found to be involved in a number of neuroinflammatory-related diseases, such as multiple sclerosis, Parkinson's disease (PD), and stroke (McKenzie et al., 2018; Li Q. et al., 2020; Zhang X. et al., 2020). Recent studies have found that the onset of depression is closely associated with neuronal pyroptosis in relevant brain regions and neuroinflammation induced by it. Herein, this study summarizes the recent research progress and thus describes the link between depression and pyroptosis, with the aim of providing new therapeutic ideas for the treatment of depression.

## Pyroptosis and Inflammation

Pyroptosis is a non-apoptotic form of cell death that is highly associated with the immunostimulant of the GSDM protein superfamily (Chen et al., 2016). In terms of molecular mechanisms, the pyroptosis mechanism can be divided into two pathways, namely, the canonical pathway mediated by caspase-1 and GSDMD, and the non-canonical pathway initiated by caspase-4/-5/-11 or caspase-3 (Wang et al., 2019). After removing the C segment of GSDM protein, the N-terminal segment forms an oligomer and a pore structure on the cell membrane (Ding et al., 2016). With the rapid formation of membrane pores (10–15 nm in diameter), intracellular ion gradients dissipate through these pores, leading to cell swelling and osmotic lysis and release of pro-inflammatory components, including IL-1β, IL-18, high mobility group box-1 (HMGB1) protein, and heat shock proteins (Cookson and Brennan, 2001).

**Figure 1:**
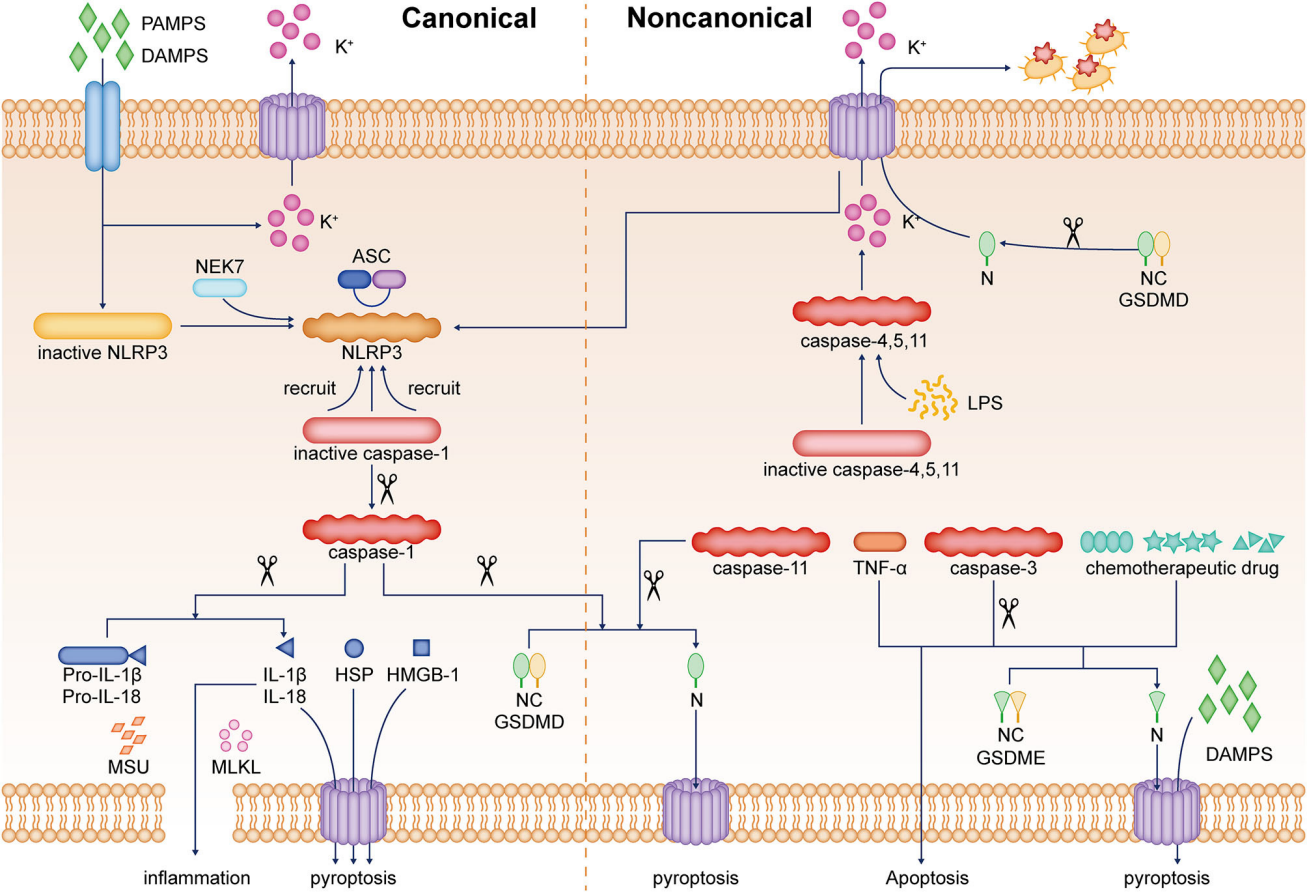
Pyroptosis is an inflammatory cell death. The mechanism of pyroptosis can be divided into two pathways, namely, the canonical pathway mediated by caspase-1 and the non-canonical pathway mediated by caspase-4/-5/-11 or caspase-3. In the canonical pathway of pyroptosis, the sensors, such as NLRP3 inflammasome and PRRs, are activated by stimuli, including PAMPs, DAMPs, etc. In addition, NLRP3 is activated by potassium ion efflux and the binding of NEK7. NLRP3 recruits pro-caspase-1 through ASC. The recruited inactive caspase-1 is activated by a self-shearing mechanism. Caspase-1 rapidly cleaves the N-terminal part of GSDMD, thus eliminating the inhibitory effect of the C-terminal end on the N-terminal part and inducing pyroptosis. In a non-canonical pathway of pyroptosis, LPS directly activates caspase-4/5/11 and cleaves GSDMD, causing GSDMD-mediated potassium ion efflux, which in turn promotes NLRP3 inflammasome activation in the canonical pathway. Other membrane-damaging substances, such as MLKL or MSU, can also induce cell membrane damage and promote IL-1β release by mediating the phosphorylation of mixed-lineage kinase structural domains. In addition, the cleavage of GSDME can convert TNF-α, chemotherapeutic drugs, and caspase-3-mediated apoptosis into pyroptosis and promote the release of DAMPs. The N-terminal segment released by GSDMD cleavage can also directly kill bacteria outside the host cell. HMGB1, high mobility group box-1 protein; HSP, heat shock protein; PAMPs, pathogen-associated molecular patterns; DAMPs, damage-associated molecular patterns; NEK7, NIMA-related kinase; ASC, adapter protein apoptotic speck-like protein; LPS, lipopolysaccharide; TNF-α, tumor necrosis factor α; MLKL, mixed-lineage kinase domain-like protein; MSU, monosodium urate crystal; PRRs, pattern recognition receptors; NLRP3, nucleotide-binding oligomerization domain (NOD)-like receptor protein 3; GSDMD, gasdermin D.

The activation of the inflammasome, such as nucleotide-binding oligomerization domain (NOD)-like receptor protein 3 (NLRP3), NLRP1b, NLRC4, absent in melanoma 2 (AIM2), and pyrin, facilitates the induction of pyroptosis and inflammation (Man et al., 2017). In the canonical model of pyroptosis, stimulus, such as pathogen-associated molecular patterns and damage-associated molecular patterns, can be recognized by NLRP3 and other inflammasome sensors and pattern recognition receptors (Liu and Lieberman, 2017; Frank and Vince, 2019). For example, in the extracellular setting, ATP is part of a molecular pattern associated with an injury that binds as a molecular distress signal to P2X7R (purinergic receptor P2X7), which forms a pore that allows immediate efflux of potassium ions and inward flow of sodium and calcium ions, ultimately leading to cellular pyroptosis (von Muecke-Heim et al., 2021). Toll-like receptor 4 (TLR4) is a pattern recognition receptor that recognizes PAMPs, including exogenous TLR4 agonists, such as bacteria, viruses, fungi, and lipopolysaccharide (LPS), whereas high mobility group box-1 (HMGB1) and heat shock proteins (HSPs) are endogenous TLR4 agonists (Zhang P. et al., 2020). These stressors indirectly activate NLRP3 by promoting potassium ion efflux and binding to NIMA-related kinase (NEK) of NLRP3, triggering its oligomerization (He et al., 2016). NLRP3 then recruits pro-caspase-1 through the adapter protein apoptotic speck-like protein containing CARD (ASC), and the recruited pro-caspase-1 is activated by its own shearing (Boucher et al., 2018). Activated caspase-1 rapidly cleaves the N-terminal part of GSDMD, eliminating the inhibitory effect of the C-terminal part connected with the N-terminal part and inducing pyroptosis (Liu and Sun, 2019). It has been shown that in a non-canonical model of pyroptosis, LPS directly activates caspase-4/5/11 and cleaves the GSDMD, but does not lead to IL-1β and IL-18 release (Shi et al., 2014; Ramirez et al., 2018). However, activation of caspase-4/5/11 can cause GSDMD-mediated potassium ion efflux, which in turn induces NLRP3 inflammasome activation in the canonical pathway with the release of IL-1β and IL-18 (Baker et al., 2015; Rühl and Broz, 2015). Furthermore, it was revealed that cleavage of GSDME could convert tumor necrosis factor α and chemotherapeutic drug-induced and caspase-3-mediated apoptosis to pyroptosis while promoting the release of damage-associated molecular patterns (Wang et al., 2017). To date, caspase-1 and caspase-11-induced formation of GSDMD N-terminal pores is considered an important prerequisite for the effective release of activated IL-1β (Kayagaki et al., 2015). A few other membrane-damaging substances, such as mixed-lineage kinase domain-like protein (MLKL) or monosodium urate crystal, can also promote IL-1β release in the absence of GSDMD by mediating the phosphorylation of mixed-lineage kinase structural domains (Conos et al., 2017; Gutierrez et al., 2017). In addition, it is shown that activated GSDMD not only induces pore formation in the host cell membrane but also kills bacteria by its pore-forming properties on the surface of bacteria. The N-terminal of GSDMD released after lysis also directly kills bacteria outside the host cell, including *Escherichia coli, Staphylococcus aureus*, and *Listeria monocytogenes* (Liu et al., 2016). Pyroptosis is beneficial to the body's defense against the invasion of foreign pathogens and the timely removal of self-damaged structures, and to maintain the normal physiological functions of various organs of the body to a certain extent. However, persistent or excessive pyroptosis and its induced neuroinflammation have now become an important pathological mechanism in many diseases (Figure 1).

## Pyroptosis, Neuron Damage, and Neurological Diseases

### Pyroptosis Emerges as an Important Pathological Mechanism in Neurological Diseases

Pyroptosis is closely associated with brain damage due to infection and hemorrhage. In LPS-stimulated rat models, increased caspase-1 activity in astrocytes and the release of large amounts of pro-inflammatory cytokines, such as IL-1β and IL-18, mediate inflammatory brain injury (Sun et al., 2019). The human immunodeficiency virus-1 envelope protein gp120 was found to cause neurological disease dependent on NLRP3-mediated pyroptosis (He et al., 2020). In a rat model of subarachnoid hemorrhage, triggering receptor expressed on myeloid cells 1 (TREM-1) further worsens neuroinflammatory injury by activating NLRP3 inflammasome-induced microglia pyroptosis. The use of LP17 would significantly ameliorate this injury by reducing the levels of GSDMD-N (Xu P. et al., 2021). In addition, in intracranial hemorrhage (ICH) mice, AC-YVAD-CMK (a selective inhibitor of caspase-1) inhibited pyroptosis and neuroinflammation by suppressing caspase-1 activation and IL-1β production, ultimately promoting the recovery of neurological function after ICH (Lin et al., 2018).

Neuronal pyroptosis is one of the important forms of ischemia-reperfusion (I/R) brain injury. In mouse I/R models, I/R induces the release of double-stranded DNA and activates AIM2 to induce neuronal pyroptosis. Knockdown of AIM2 and the use of AC-YVAD-CMK can attenuate I/R injury (Li et al., 2019). In a mouse model of middle cerebral artery occlusion (MCAO), long non-coding RNA maternally expressed gene 3 promotes AIM2-induced pyroptosis by inhibiting miR-485, thus enhancing cerebral I/R-induced injury (Liang et al., 2020). In a rat model of liver I/R injury, serum-derived exosomes mediate neuronal pyroptosis and induce reactive oxygen species (ROS) and malondialdehyde production in hippocampal and cortical regions of the brain (Zhang et al., 2019). Increased lncRNA-H19 in I/R can lead to disruption of NLRP3/6 inflammasome homeostasis and microglial and neuronal pyroptosis (Wan et al., 2020). I/R-injured intestinal endothelial cells cause cortical neuronal necrosis or pyroptosis by releasing exosome miRNAs (Hsu et al., 2020). The expression of low-density lipoprotein receptor (LDLR) is reduced in I/R injury. LDLR deficiency caused by I/R can enhance the NLRP3/caspase-1/GSDMD signaling pathway leading to severe neuronal pyroptosis. The use of the NLRP3 inhibitor CY-09 can prevent LDLR deficiency-induced pyroptosis (Sun et al., 2020).

The onset and progression of pyroptosis and PD are closely related. α-Synuclein was found to induce activation of NLRP3 inflammasome in mouse microglia, thereby inducing sustained dopaminergic neuronal damage and PD progression (Gordon et al., 2018). In contrast, the NLRP3 inhibitor MCC950 (a specific small-molecule inhibitor of the NLRP3 inflammasome) was found to ameliorate isoflurane-induced pyroptosis while improving cognitive impairment (Fan et al., 2018). AIM2 inflammasome was found to promote long-term cognitive impairment and memory loss induced by chronic cerebral hypoperfusion (Poh et al., 2021). In a mouse model of MCAO/reperfusion-induced post-stroke cognitive impairment, AIM2 expression was significantly elevated in the hippocampus and cortex relative to controls. AIM2 knockdown and the use of AC-YVAD-CMK improved cognitive function and inhibited hippocampal volume reduction (Kim et al., 2020). Sepsis-associated encephalopathy can lead to long-term cognitive impairment in patients. It was found that the levels of NLRP3 and caspase-1 positive cells, as well as pyroptosis and inflammation-related proteins, were significantly increased in the hippocampus of the brains of mice with sepsis-associated encephalopathy, and the use of MCC950 and AC-YVAD-CMK significantly improved cognitive function loss (Fu et al., 2019).

Recent studies have shown that pyroptosis is associated with the development of Alzheimer's disease (AD). Amyloid β, a major pathogenic factor in AD, was found to cause pyroptosis in mouse cortical neurons by activating the NLRP3-caspase-1 signaling pathway and promoting the expression of GSDMD protein (Han et al., 2020). In addition, pyroptosis can mediate brain I/R-induced neurotoxic amyloid β aggregation and blood–brain barrier and lymphatic system dysfunction (Lyu et al., 2021). This will further lead to the deterioration of AD.

### Pyroptosis Is a Potential Target for the Treatment of Neurological Diseases

Gradually accumulating evidence suggests that multiple anti-pyroptosis approaches can have anti-inflammatory and neuroprotective effects. Physical hypothermia treatment can effectively inhibit ischemic brain injury. Hypothermia significantly increases the activity of hippocampal neuronal cells exposed to hypoxia/reoxygenation by inhibiting the expression of pyroptosis-related proteins, such as NLRP3, ASC, caspase-1, and GSDMD. In addition, a similar effect was produced by using MCC950 (Diao et al., 2020).

A variety of chemical agents have been found to have anti-pyroptosis and therapeutic effects on neurological diseases. In a mouse traumatic brain injury model, rapamycin activates autophagy to downregulate the expression of pyroptosis-related proteins and IL-13, and inhibits the JAK-1-STAT-1 signaling pathway, ultimately inhibiting neuronal pyroptosis caused by injury (Gao et al., 2020). In a mouse model of Huntington's disease, olaparib increased survival and improved neurological deficits by inhibiting inflammasome activation and pyroptosis (Paldino et al., 2020). Atorvastatin was found to inhibit neuronal pyroptosis and neuroinflammation by suppressing NLRP1 expression, thereby improving neurological damage caused by early subarachnoid hemorrhage (Chen J. et al., 2021).

A number of biological molecules have anti-neuronal pyroptosis activity. Melatonin inhibits caspase-1 expression through upregulation of miR-214-3p, which ultimately inhibits neuronal pyroptosis and protects neurons from the toxic effects of high glucose concentrations (Che et al., 2020). In addition, plasma exosomes pretreated with melatonin inhibited toll-like receptor (TLR) 4/nuclear factor-kappa B (NF-κB) to reduce neuronal pyroptosis induced by cerebral ischemia, ultimately reducing ischemic infarct volume and promoting neurological recovery (Wang et al., 2020). In rat cortical neurons subjected to oxyglucose deprivation and reoxygenation, osteocalcin reduces proline hydroxylase1 and inhibits the catabolic activation of GSDMD, thereby promoting the conversion of neuronal glucose metabolism to pentose phosphate and inhibiting neuronal pyroptosis (Wu et al., 2020). Insulin-like growth factor 1 is a specific target of miR-129. Studies have shown that inhibition of miR-129 and thus the upregulation of insulin-like growth factor 1/glycogen synthase kinase-3 signaling pathway can improve the effects of neuronal death and cognitive impairment caused by neuronal pyroptosis (Wang F. et al., 2021).

**Figure 2:**
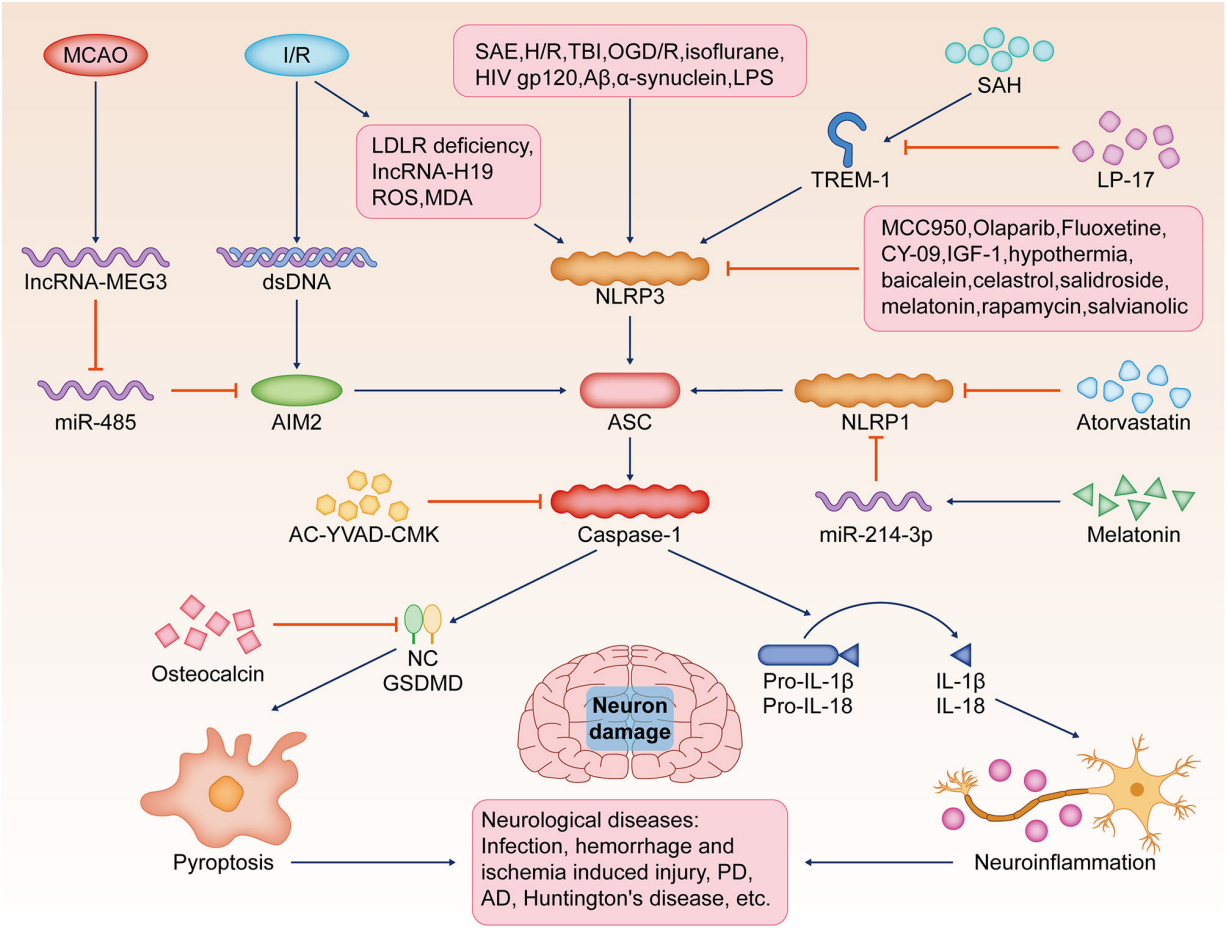
Pyroptosis, neuron damage, and neurological diseases. Pyroptosis is a potential target for the pathological mechanism and treatment of various neurological diseases, such as brain infection, hemorrhagic/ischemic injury, Huntington's disease, AD, or PD. SAE, H/R, TBI, OGD/R, isoflurane, Aβ, LPS, or HIV-gp120 stimulate NLRP3-mediated pyroptosis, resulting in increased caspase-1 activity in the brain and the release of a large number of pro-inflammatory cytokines, such as IL-1β and IL-18. I/R induces the release of dsDNA and activates AIM2 to induce neuronal pyroptosis. In addition, reduced LDLR, IncRNA-H19, ROS, and MDA lead to severe neuronal pyroptosis by activating the NLRP3/caspase-1/GSDMD signaling pathway. SAH, TREM-1-activated microglial pyroptosis induced by NLRP3 inflammasome, and the use of TREM-1 inhibitor LP17 significantly ameliorated this damage. MCAO, induced by lncRNA MEG3, promotes AIM2-induced pyroptosis by sponging miR-485. NLRP3 inhibitor MCC950, olaparib, fluoxetine, CY-09, IGF-1, hypothermia, baicalein, celastrol, salidroside, melatonin, rapamycin, and salvianolic acid significantly reduce neuronal injury by inhibiting pyroptosis. AC-YVAD-CMK inhibits caspase-1 activation. Atorvastatin and melatonin inhibit NLRP1 expression. Osteocalcin inhibits the activation of GSDMD, thereby inhibiting pyroptosis. SAE, sepsis-associated encephalopathy; H/R, hypoxia/reoxygenation; TBI, traumatic brain injury; OGD/R, oxyglucose deprivation and reoxygenation; Aβ, amyloid β; LPS, lipopolysaccharide; NLRP3, the nucleotide-binding oligomerization domain (NOD)-like receptor protein 3; (IL)-1β, interleukin; AD, Alzheimer's disease; PD, Parkinson's disease; I/R, ischemia-reperfusion; dsDNA, double-stranded DNA; AIM2, the absent in melanoma 2; LDLR, the low-density lipoprotein receptor; ROS, reactive oxygen species; MDA, malondialdehyde; GSDMD, gasdermin D; SAH, subarachnoid hemorrhage; TREM-1, trigger receptor expressed on myeloid cells 1; MCAO, middle cerebral artery occlusion; lncRNA MEG3, long non-coding RNA maternally expressed gene 3; IGF-1, insulin-like growth factor 1; ASC, the adapter protein apoptotic speck-like protein containing CARD.

A variety of natural plant extracts exert significant anti-pyroptosis effects. In rats with acute spinal cord injury, celastrol exerts anti-inflammatory and neuroprotective effects by inhibiting the expression of pyroptosis-related proteins and the release of inflammatory cytokines (Dai et al., 2019). In N-methyl-4-phenyl-1,2,3,6-tetrahydropyridine-induced PD mice, salidroside inhibited thioredoxin-interacting protein/NLRP3/caspase-1 signaling by suppressing the pathway, thereby inhibiting neuronal pyroptosis and neuroinflammation (Zhang X. et al., 2020). It was shown that salvianolic acid significantly improved brain I/R injury by promoting microglia conversion to the M2 phenotype and inhibiting NLRP3 activation-induced microglia pyroptosis (Ma et al., 2021). Baicalein reversed N-methyl-4-phenyl-1,2,3,6-tetrahydropyridine-induced motor dysfunction, dopaminergic neuronal damage, and neuroinflammation by inhibiting the NLRP3/caspase-1/GSDMD signaling pathway (Rui et al., 2020). Therefore, baicalein may be a potential drug for PD treatment (Figure 2).

## Most Recent Evidence of the Relationship Between NLRP3 Inflammasome and Depression

### NLRP3 Inflammasome Deteriorates Depression-Like Behaviors by Triggering Neuroinflammation

Clinical and preclinical studies suggest inflammasome as a potential therapeutic strategy for the treatment of inflammation-related psychiatric disorders (Kwon and Cheon, 2021). NLRP3 inflammasome activation drives the progression of depression and is detrimental to post-stress recovery (Pandey et al., 2021; Yang et al., 2021). Fluoxetine, a clinical antidepressant licensed by the Food and Drug Administration, has been found to bind and inhibit NLRP3-ASC inflammasome (Ambati et al., 2021). In cigarette smoke-induced chronic obstructive pulmonary disease (COPD) and depression mouse models, cigarette smoke-induced glucocorticoid resistance was associated with NLRP3 inflammasome activation-induced inflammatory cascade responses. Long-term fluoxetine treatment reversed these alterations (Deng et al., 2019). In the chronic unpredictable mild stress (CUMS) mouse model, human umbilical cord mesenchymal stromal cells reduced neuronal complement C3 receptors and thereby attenuated NLRP3 activation-induced neuroinflammation and ultimately ameliorated CUMS-induced depression-like behavior (Li J. et al., 2021). Acupuncture in physiotherapy can exert antidepressant effects by reducing the levels of NLRP3 inflammasome and inflammatory factors in the prefrontal cortex of depression rats (Li X. et al., 2021). In addition, B-cell scavenger CD36 receptors are associated with cytotoxicity and neuroinflammation. Relative to wild-type mice, CD36(-/-) mice have downregulated NLRP3 inflammasome signaling and attenuated depression-like manifestations induced by chronic stress (Bai et al., 2021).

### Experimental Agents Ameliorate Depression-Like Behaviors by Suppressing NLRP3

Various experimental reagents can ameliorate depression-related symptoms by inhibiting the NLRP3 inflammasome. NU9056 can reduce LPS-induced cognitive impairment and affective disorders in mice by inhibiting NLRP3 inflammasome activation (Chen L. et al., 2021). Simvastatin inhibits the activation of hippocampal microglia, suppresses the expression of P2X7 receptors, TLR2, and TLR4, and inhibits the activation of the NLRP3 inflammasome, thereby reducing the levels of IL-1β and IL-18 and exerting some antidepressant effects (Menze et al., 2021). Disturbance of Ca (2+) homeostasis in patients with major depressive disorder induced by abnormal activation of P2X7 receptors can also lead to the activation of caspase-1 that is not dependent on inflammasome and subsequent inflammation (Garrosa-Jiménez et al., 2021). Rapamycin increases catalase and superoxide dismutase and reduces NLRP3 gene expression, thereby suppressing pentylenetetrazole-induced anxiety and depression-like manifestations (Aghaie et al., 2021). Higher levels of endothelin (ET)-1 are associated with the activation of the NLRP3 pathway, and the brain ET-1 exerts effects through its primary receptor, ET_B_R. Dapagliflozin (DaPa) plays an antidepressant role by regulating the NLRP3/ET-1/ET_B_R/brain-derived neurotrophic factor/zonula occludens 1 axis, enhancing blood–brain barrier integrity and neuroplasticity (Muhammad et al., 2021). Transient receptor potential vanilloid 4 levels were significantly increased in the hippocampus of the LPS-induced depression model in mice. Transient receptor potential vanilloid 4 inhibitor HC067047 decreased the activation of hippocampal astrocytes and microglia, reduced CaMKII-NLRP3 inflammasome expression, and increased the expression of the hippocampal neurogenesis marker DCX, thereby exerting antidepressant effects (Li W. et al., 2021). Memantine blockade of N-methyl-D-aspartate receptors reduces NLRP3 inflammasome and astrocyte activation, and calain-1 expression, ultimately inhibiting LPS-induced depressive-like behavior (Song et al., 2021). The mechanism of the action of ketamine to improve anxiety-like performance is concentration-dependent, and the antidepressant-like effects of low concentration of ketamine are not dependent on NLRP3 (Camargo et al., 2021a,b).

### Natural Product Ingredients Exert Significant Anti-depression Effects via Suppressing NLRP3

Various components of natural products showed significant antidepressant activity in animal model experiments. The oligosaccharides of *Morinda officinalis* reduced NLRP3 expression, thereby alleviating depression-like behavior in post-stroke depression (PSD) rats. It was shown that the negative regulation of NLRP3 by the oligosaccharides of *Morinda officinalis* was associated with the inhibition of the NF-κB signaling pathway (Li Z. et al., 2021). Ganoderic acid A inhibits the activation of NLRP3 by modulating the prefrontal cortical bile acid receptor farnesoid X receptor in mice, thus exerting antidepressant effects (Bao et al., 2021). Catalpol, phosphodiesterase-4 inhibitors, and Kai Xin San have been demonstrated to improve CUMS-induced depression-like behavior in mice through NLRP3 activation (Wang Y. et al., 2021; Xie et al., 2021; Yu et al., 2021). Albiflorin may alleviate depression-like behavior induced by chronic constriction injury of the sciatic nerve by inhibiting the activity of the NLRP3 inflammasome (Liu et al., 2021). Saponins from *Panax japonicus* treatment may alleviate high-fat diet-induced depression-like behavior by inhibiting NLRP3 inflammasome (Wang J. et al., 2021). NLRP3 may also mediate the regulation of depression by the gut–brain axis (Hao et al., 2021; Zhu et al., 2021). Fecal microbiota transplantation inhibited the activation of Iba-1 (an essential hallmark of microglia and astrocytes cells)-positive microglia and glial fibrillary acidic protein-positive astrocytes and decreased NLRP3 expression in the frontal cortex and hippocampus, ultimately improving depressive-like behavior (Rao et al., 2021).

### Does NLRP3 or Other Inflammasome Play a Dual Role in Depression Regulation?

**Figure 3:**
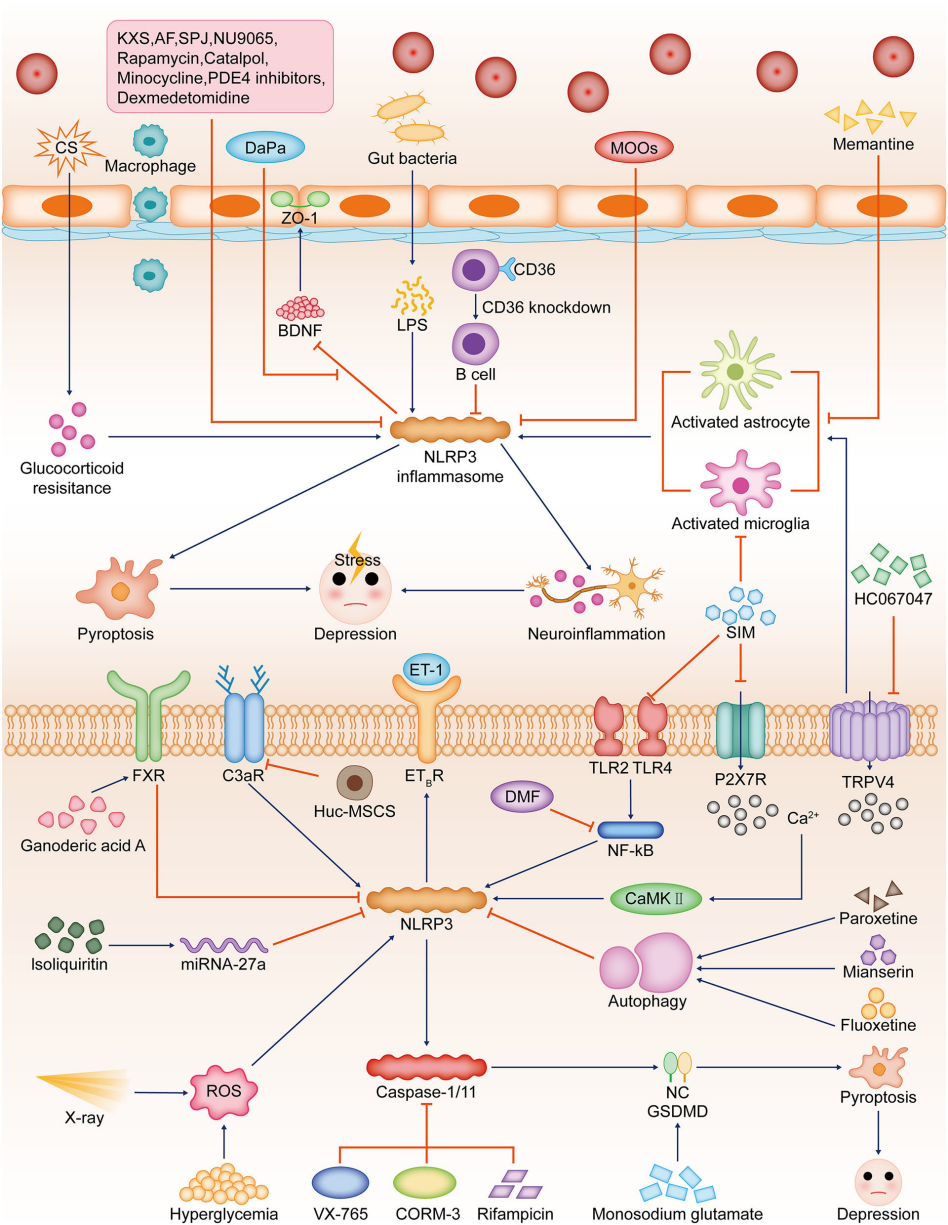
Relationship between NLRP3-dependent pyroptosis and depression. In different models of stress-induced depression, NLRP3 inflammasome activation can worsen depression-like behaviour by inducing pyroptosis and triggering neuroinflammation. Hyperglycemia and X-ray can induce ROS production, which leads to NLRP3 inflammasome activation in hippocampal neurons. CS-induced glucocorticoid antagonism promotes NLRP3 inflammasome activation. hUC-MSCs reduce C3aR, which attenuated NLRP3 activation. SIM inhibits hippocampal microglia and NLRP3 inflammasome activation by suppressing the expression of P2X7R, TLR2, and TLR4. Ca(2+) disturbance caused by P2X7R and TRPV4 dysfunction can activate CaMKII and then promote NLRP3 inflammasome activation. Monosodium glutamate can upregulate GSDMD. These will lead to NLRP3-dependent pyroptosis and depression-like manifestations. NLRP3 inflammasome signaling is downregulated in CD36(-/-) mice. The TRPV4 inhibitor HC067047 inhibits hippocampal glial cell activation and NLRP3 inflammasome expression. NLRP3 activation promotes ET-1 release and thus reduces ET_B_R expression. DaPa maintains the blood–brain barrier integrity by regulating the NLRP3/ET-1/E _B_ TR/BDNF/ZO-1 axis. Ganoderic acid A inhibits NLRP3 activation by upregulating FXR in mice. NU9065, rapamycin, memantine, MOOs, catalpol, PDE4 inhibitors, KXS, AF, SPJ, minocycline, and dexmedetomidine can also inhibit NLRP3 activation. Dexmedetomidine can also inhibit the activation of NLRP3 inflammasomes. The antidepressants fluoxetine, paroxetine, and mianserin induce autophagy and thus inhibit NLRP3 activation. Isoliquiritin inhibits NLRP3 activation by upregulating miRNA-27a. DMF reduces NLRP3 levels by inhibiting the NF-κB signaling pathway. VX-765, CORM-3, and rifampicin can reduce depression by inhibiting caspase-1/11. Downregulation of NLRP3-dependent pyroptosis-related pathways alleviates depression-like behaviors. CS, cigarette smoke; CUMS, chronic unpredictable mild stress; hUC-MSCs, human umbilical cord mesenchymal stromal cells; C3aR, neuronal complement C3 receptors; SIM, simvastatin; ET, endothelin; DaPa, dapagliflozin; BDNF, brain-derived neurotrophic factor; ZO-1, zonula occludens 1; TRPV4, transient receptor potential vanilloid 4; MOOs, *Morinda officinalis* oligosaccharides; FXR, farnesoid X receptor; AF, albiflorin; SPJ, saponins from *Panax japonicus*; DMF, dimethyl fumarate; PDE4, phosphodiesterase-4; KXS, Kai Xin San.

Numerous studies have shown that activation of NLRP3 will contribute to worsening depression-like behavior. Some studies have found that deletion of some types of inflammasome also leads to anxiety-like manifestations, and conversely, low-level activation suppresses depression. The deletion of NLRP3 leads to synaptic transmission impairment, anxiety-like manifestations, and labored fear learning, suggesting an important role of NLRP3-mediated low-level inflammation in memory consolidation (Komleva et al., 2021). AIM2 recognizes damaged genes and contributes to neuronal pyroptosis, thereby shaping the development of the central nervous system, and the absence of this receptor would lead to anxiety-like behaviors in mice (Lammert et al., 2020). However, the number of related studies is still insufficient and further experimental exploration is needed (Figure 3).

## NLRP3 Inflammasome: The Convergence Between Pyroptosis and Depression

### NLRP3-Dependent Pyroptosis Plays a Pivotal Role in Depression Pathogenesis

Pyroptosis plays an important role in the pathogenesis and progression of depression. A clinical study shows that overexpression of IL-1/IL-6 and downregulation of mevalonate kinase in the monocytes of depressed patients with childhood adversity may induce a cellular transition from premature aging, inflaming to more severe pyroptosis (Simon et al., 2021). In pathological situations, gut microbes and their antigenic secretions mediate the development of neuropsychiatric disorders, including schizophrenia, depression, anxiety, and bipolar disorders, by promoting pyroptosis induced by the activation of microglia inflammasome (Rutsch et al., 2020). Loss of astrocytes in the hippocampus of the brain is one of the most important features of depressed patients and depressed mice. It has been shown that astrocyte pyroptosis in depressed mice is associated with the loss of hippocampal astrocytes. Selective serotonin reuptake inhibitors can attenuate pyroptosis induced by chronic mild stimulation. On the contrary, knockdown of GSDMD, caspase-1, and astrocyte NLRP3 inflammasome genes attenuated depression-like manifestations (Li S. et al., 2021). In streptozotocin-induced diabetic mice, hyperglycemia-induced ROS production, which induced NLRP3 inflammasome activation in hippocampal neurons and an increase in GSDMD-N, ultimately leading to neuronal pyroptosis in the hippocampal region and depressive-like manifestations in mice (Li et al., 2020). Monosodium glutamate can induce depressive-like manifestations in mice by upregulating caspase-1, GSDMD, IL-1β, IL-18, and NLRP3. In a rat model of depression induced by monosodium glutamate, it was found that both minocycline and VX-765 (a caspase-1-specific inhibitor) improve the depressive-like performance of rats. Among them, the use of minocycline significantly reduced both HMGB1/the receptor for advanced glycation end products/NLRP3 and GSDMD-induced hippocampal neuronal pyroptosis. In contrast, VX-765 inhibited only GSDMD signaling, suggesting a key role of GSDMD-dependent pyroptosis in the progression of depression (Yang et al., 2020). As the application of X-rays becomes more widespread, the chance of radioactive brain injury also rises. The main manifestations of brain injury induced by radiation include cognitive dysfunction and neuroinflammation, which severely affect human health (Xu et al., 2021). Studies have shown that X-rays induce the production of ROS in the prefrontal cortex in a dose-dependent manner, which leads to depression-like behavior in mice. In addition, X-ray exposure will promote HMGB1 expression, which activates NLRP3-dependent pyroptosis and induces neuroinflammation, ultimately leading to neuronal loss and depressive-like behavior. In contrast, the HMGB1 inhibitor glycyrrhizin reversed the depression-like behavioral changes induced by X-rays (Xu et al., 2021).

### Pyroptosis Inhibitors Promote Alleviation of Depressive Behaviors

Some antidepressants have potential anti-pyroptosis activity. In animal models of stress-induced depression and depressed patients, antidepressants, including fluoxetine, paroxetine, and mianserin, induce autophagy, thereby promoting a decrease in serum IL-1β and IL-18 levels and inhibiting the expression of NLRP3 and IL-1β (Alcocer-Gómez et al., 2017). In addition, several drugs with antidepressant activity have been found to exert neuroprotective and antidepressant effects through their antiangiogenic properties. Rats with hemorrhage shock and resuscitation (HSR) exhibit enhanced depression and anxiety-like manifestations, accompanied by increased pyroptosis of amygdala neurons and astrocytes. Dexmedetomidine, an α2-adrenoceptor agonist, exerts a potential antidepressant effect by inhibiting astrocyte pyroptosis (Sun et al., 2019). CORM-3 ameliorates HSR-induced neuronal pyroptosis by downregulating astrocyte IL-18 expression and exerts a neuroprotective effect in HSR (Zhang D. X. et al., 2020). Further studies found that CORM-3-injected rats exhibited less amygdala neuronal pyroptosis and apoptosis, and upregulated phosphorylation of the extracellular signal-regulated kinase 1/2. This neuroprotective effect was partially reversed by a specific inhibitor of protein kinase G (PKG), suggesting that the effect of CORM-3 on improving depression and anxiety-like performance is related to the PKG-extracellular signal-regulated kinase 1/2 signaling pathway (Li Y. et al., 2020).

Expression of miRNA-27a was found to be significantly reduced in serum obtained from depressed patients and in the serum and hippocampus of mouse models of depression induced by LPS or chronic social defeat stress. It was shown that isoliquiritin administration decreased LPS and adenosine triphosphate-induced NLRP3 inflammasome activation and pyroptosis-related protein expression, while miRNA-27a inhibitors significantly reversed this effect (Li Y. et al., 2021). This suggests that isoliquiritin has an anti-neuronal pyroptosis and antidepressant effect by upregulating miRNA-27a. It was shown that dimethyl fumarate can reduce IL-1β, IL-18, caspase-1, and NLRP3 levels through the Nrf2/NF-κB signaling pathway, thus inhibiting LPS-induced microglial pyroptosis and disease manifestations in LPS-challenged depression mice (Tastan et al., 2021). In the lisproin-induced depression mouse models, paeoniflorin improved motility and abnormal synaptic plasticity alterations in the hippocampus in tail suspension experiments and forced swimming experiments. Further studies revealed that paeoniflorin inhibited rifampicin-induced caspase-11-dependent pyroptosis of hippocampal microglia in mice, thereby suppressing neuroinflammation and exerting antidepressant effects (Tian D. D. et al., 2021) (Figure 3).

## Discussion and Remark

Numerous studies have shown that NLRP3 activation is a key factor in the pathogenesis of depression, and neuroinflammation induced by NLRP3 activation was once thought to be an important causative factor for depression. Studies in recent years have shown that NLRP3-dependent cellular pyroptosis is also an important mechanism of depression-related injury. This article reviews the role of pyroptosis in various neurological diseases and depression. Anti-pyroptosis treatment exerted significant neuroprotective effects in animal models of various neurological disorders, including depression. In depression-related animal model experiments, multiple antidepressant agents exhibited anti-pyroptosis activity, and in addition, multiple agents targeting pathways related to the regulation of pyroptosis have been shown to have antidepressant activity. Physical cryotherapy, chemical molecules, and natural products targeting the pathways related to the regulation of pyroptosis may be persistently effective antidepressant treatment strategies. These findings suggest that NLRP3-dependent pyroptosis-related pathways may become new targets for the treatment of depression.

Yet, there are still shortcomings in the current studies. First, current studies on pyroptosis and neurological diseases are limited to NLRP3 and AIM2 inflammasomes, and the link between other types of pyroptosis-related inflammasomes, such as NLRC4, NLRP6, and depression, needs to be further investigated and elucidated. Second, current studies have shown that GSDMA, GSDMB, GSDMC, GSDMD, and deafness autosomal dominant type 5 can induce the formation of pore channels in the cell membrane and thus induce the onset of cellular pyroptosis (Shi et al., 2017). However, the upstream regulatory mechanisms of GSDM family members other than GSDMD remain unclear, and their relationship with depression remains to be elucidated. Third, more mechanistic understanding is needed on how stress-induced changes in neuronal intracellular pathways lead to the development of pyroptosis and then depression. Forth, in addition to the hippocampus, amygdala, and prefrontal cortex, it has recently been found that lateral habenula is also related to the regulation of depression (Hu et al., 2020). In the animal models of depression induced by various stress factors, the spatiotemporal profile of pyroptosis of different cells in different brain regions needs to be further measured.

The above-mentioned challenges suggest that the mechanism of interaction between pyroptosis and depression still has much room for research. At present, while targeting NLRP3-dependent pyroptosis to treat depression is emphasized, it is also important to note that appropriate inflammasome activation-induced inflammation may be beneficial for neuro-environmental homeostasis. Both excessive activation and complete inhibition of inflammasome will lead to the worsening of depressive symptoms. This speculation is also supported by the evidence that the microglia decline in the late stage of depression aggravates the depression, but inducing microglia activation at this time improves the symptoms of depression significantly (Jia et al., 2021). Therefore, simultaneous qualitative and quantitative modulation of NLRP3-dependent pyroptosis may be crucial to achieving the desired therapeutic outcome.

## Author Contributions

TW designed this study. TW, XL, and MF wrote the manuscript. PL and XG prepared figures. TW and WG critically revised the manuscript for important intellectual content. All authors read and approved the final manuscript and agree to be accountable for all aspects of this work.

## Conflict of Interest

The authors declare that the research was conducted in the absence of any commercial or financial relationships that could be construed as a potential conflict of interest.

## Publisher's Note

All claims expressed in this article are solely those of the authors and do not necessarily represent those of their affiliated organizations, or those of the publisher, the editors and the reviewers. Any product that may be evaluated in this article, or claim that may be made by its manufacturer, is not guaranteed or endorsed by the publisher.
